# Supplementary material for: Practitioners’ perspectives on implementation of acute virtual wards: A scoping review
Source: PLOS Digit Health. 2025 May 9;4(5):e0000858. doi: 10.1371/journal.pdig.0000858 (PMC12063850; doi:10.1371/journal.pdig.0000858)
Supplement: Attachment — Submitted filename: 24 00573 review response.docx [file pdig.0000858.s002.docx]

Thank you for the opportunity to review this manuscript. The concept of virtual care and specifically ‘virtual wards’ is evolving and generating greater interest within health care systems. I believe this research is timely and important. The edits and comments are intended to strengthen this work and enhance clarity, accuracy, and reader ease of comprehension.

Line 51- 53 Are you stating that the increase in adults over the age of 80 years is responsible for ER and hospital over-crowding? Please revise with consideration given to the multiple contributors to hospital/ emergency use. This appears to be an ageist perspective on health systems utilization.

Line 55-56 Clarity and greater description would be helpful and would contribute to a more comprehensive understanding of health service challenges

Line 60-63 My understanding is that virtual care also exists within acute care settings and not isolated within home settings. Please confirm and consider revision

Line 70 “fully embed service models” -please provide clarity to this suggestion; are you referring to virtual wards?

Line 74-75 you make a claim that “…VW programme exemplifies the solutions promoted by HSE’s ‘Telehealth Roadmap 2024-27’ strategy - please outline how VWs will address the suggested strategies included in the Roadmap.

Line 78 and throughout the manuscript please use consistent terms or define roles such as “clinician” -how are you defining practitioners / clinicians?

Line 81-82 you state “Studies aimed at understanding the attitudes of those at the centre of implementation have been emphasized as a crucial component of the implementation process…” Consider re-phrasing; most successful implementation projects acknowledge the importance of an interdisciplinary team. Clinicians would be an important stakeholder as the end user.

Line 94-95 Please clarify; do you mean that a study protocol was not generated as a separate manuscript?

Line 142 Additional detail is needed for data analysis; how many researchers analyzed the data; what was your process to facilitate consistent analyses among researchers; how did you resolve differences; describe the background and experience among researchers contributing to the analysis - who had experience working with VW; what disciplines contributed (engineering, nurse, physician, IT...). Was the analysis inductive/deductive or both; did you have an initial code book to direct your analysis; did you use an implementation theory or framework to guide this analysis....

Line 163-164 Please provide clarity and revise; are you suggesting broadly that all staff (who is 'staff'?) reported satisfaction (satisfaction with what). And this was supported by one reference?

Line 164-165 See my comment above; these statements need to be tempered / contextualized

Line 181 “resource allocation” - what resources (do you mean people?)are you referring to?

Line 188-189 How did you know administration was supportive or not? Please review for clarity

Line 204-207 you state: “AVWs offer system-wide benefits, such as reduced emissions, cost savings, and shorter hospital stays (18, 27, 34). However, concerns about climate impact from vehicle use persist, with recommendations for electric vehicles (18). Financial sustainability remains a challenge, requiring public funding and regional support”; these are broad statements that require additional description/ ‘evidence’ for readers to fully comprehend and appreciate the intended.

Line 215 regarding safety and the use of alarm devices resulting in increased confidence; please provide additional insight into ‘lone workers’ and the purpose of home visits within a VW model of care; please address whose confidence is impacted - the health care organization or the provider or both? What is the scope of this concern?

Line 216-217 you state “Technology such as GPS tracking and recording devices may enhance safety”. Please consider the alternative that such tracking may elevate the experience of surveillance among providers or workers or … and this may have implications for enhanced safety but also for lack of privacy.

All results tables – please revise the title to include VW

Line 229-230 would you consider the clinical experience expressed here to be unique to only within VW models of care?

Line 234 you claim ‘…the need for active patients and roles” ; provide additional detail to support reader comprehension of this claim – describe how patients need to be ‘active’ etc.

Line 235 you “…more intimate environment…” ; do you mean their home context or lived environment? Please provide clarity

Line 280-281 requires additional description/ information

Line 311-312 consider caution in the broad statement / claim made here - the literature is limited and this is based on one or few studies

Line 382 “methodological considerations” appears to be your limitations section – please revise.
