# Supplementary material for: Practitioners’ perspectives on implementation of acute virtual wards: A scoping review
Source: PLOS Digit Health. 2025 May 9;4(5):e0000858. doi: 10.1371/journal.pdig.0000858 (PMC12063850; doi:10.1371/journal.pdig.0000858)
Supplement: Attachment — Submitted filename: Rebuttal file.docx [file pdig.0000858.s004.docx]

**Rebuttal file**

| **Editor** | |
| --- | --- |
| **Comments** | **Response** |
| We have amended your Competing Interest statement to comply with journal style. We kindly ask that you double check the statement and let us know if anything is incorrect. | Happy with the competing interest statement |
| Figure 2: please (a) provide a direct link to the base layer of the map (i.e., the country or region border shape) and ensure this is also included in the figure legend; and (b) provide a link to the terms of use / license information for the base layer image or shapefile. We cannot publish proprietary or copyrighted maps (e.g. Google Maps, Mapquest) and the terms of use for your map base layer must be compatible with our CC-BY 4.0 license. | Figure legend removed.  The information has been substituted in the paragraph “The 18 studies included were from six countries including Australia (n=1), Canada (n=5), Italy (n=1), Spain (n=1), the United States (n=4) and United Kingdom (n=5) and one study comprising Australia and United Kingdom” (Line 166-168) |
| Many of the findings are vague. Please be more specific. For example, “Engagement and active support from hospital specialists (such as cardiology) was also found to be essential in promoting referrals to VW.” What kind of engagement? What type of support? I find the contents in section 4(a), "Referral guidelines," particularly unclear. These are just examples. It would be great if the authors could go through the findings and discussion to identify content and concepts that are too general and present them more specifically. | Amendments made.  Line 313 -319 “Additionally, direct collaboration with hospital specialists, particularly cardiologists, played a critical role in promoting referrals to VW (34). This engagement included cardiologists providing clear referral criteria during multidisciplinary team meetings, conducting informational workshops for referrers, and offering ongoing case-based guidance to ensure appropriate patient selection. Services should develop clear inclusion and exclusion criteria to manage capacity, maintain activity and offer training sessions for appropriate referring.” |
| Please provide a conclusion section to present take-home messages or conclusion statements. | Conclusion added  Line 449-455  “VWs offer an alternative to traditional inpatient care, with high levels of satisfaction reported by both healthcare staff and patients. However, successful implementation depends on robust service design, clear referral pathways, and the integration of patient and caregiver perspectives. Challenges related to quality of care, technology, and training must be addressed to optimize outcomes. Future research should explore long-term patient outcomes, caregiver experiences, and disease-specific VW models to strengthen the evidence base and inform best practices.” |

| **Reviewer 1** | |
| --- | --- |
| **Comments** | **Response** |
| the evidence behind virtual wards is evolving and is less than robust - how is this likely to impact the opinions of health care staff? | Lines 347-348 in discussion and lines 436-438 in implications for research, education and practice explains the robustness.  “However, our scoping review also identifies several challenges and considerations associated with the implementation of acute virtual wards.”  “Research in this area is evolving rapidly with primary studies becoming available, ensuring that the implementation of services is informed by evidence is a priority” |
| How representative are healthcare staff who are part of this research review? | Lines 427-433 outlines the stakeholder representatives.  “Most of the articles included in this review were from Western regions (especially UK, Europe, the United States and Australia) with robust healthcare systems. Hence, findings may not be representative of those in other countries and cultures. Additionally, this scoping review examined a diverse set of stakeholders’ perspectives (e.g., healthcare professionals, administrators) of virtual wards in different contexts (e.g., rehabilitation, cardiovascular, or frailty units) that may not represent discipline- or disease-specific needs of the stakeholders.” |
| Given the significant heterogeneity of studies identified and how many are excluded how robust are the conclusions? | This may have resulted in the omission of relevant insights. These factors may limit the generalizability and strength of our findings, but the students were identified subject to a strong criteria and framework.  Lines 413-414 and 416-418  “Although we used the Arksey and O’Malley’s (15) six-step methodological framework, a few limitations should be considered when interpreting the findings of this review.”  “We also did not assess the quality of studies, as the focus was on mapping the evidence.  Another limitation includes the exclusion of papers that did not directly address acute care in VWs” |
| It is difficult to pick out the key messages in the discussion - please could this be revised? | Discussion revised.  Amended Lines 354-365 Implementation, Lines 367-381 Quality of care,  Lines 385-397 Technology and communication,  Lines 399-409 Training and awareness  to avoid redundancy. |

| **Reviewer 2** | |
| --- | --- |
| **Comments** | **Response** |
| Line 51- 53 Are you stating that the increase in adults over the age of 80 years is responsible for ER and hospital overcrowding? Please revise with consideration given to the multiple contributors to hospital/ emergency use. This appears to be an ageist perspective on health systems utilization. | Lines 51-55  “In Ireland, there is an increased risk of hospital admission largely due to demographic growth, population ageing and chronic disease, as well as other factors including seasonal surges in respiratory illnesses and health service capacity limitations (2). These factors combined lead to more emergency department (ED) presentations and hospital overcrowding, with hospitals working at full capacity.” |
| Line 55-56 Clarity and greater description would be helpful and would contribute to a more comprehensive understanding of health service challenges | Lines 55-58  “This is culminating in sustained and recorded pressure on ED services. The associated ED ‘overcrowding’ is linked to the inefficient use of resources, increased patient risk and a challenging work environment for staff” |
| Line 60-63 My understanding is that virtual care also exists within acute care settings and not isolated within home settings. Please confirm and consider revision | We are applying the NHS definition of Virtual Wards in this study. NHS defines VW as **‘**a safe and efficient alternative to NHS bedded care that is enabled by technology…virtual wards support patients who would otherwise be in hospital to receive the acute care, monitoring and treatment they need in their own home’ |
| Line 70 “fully embed service models” -please provide clarity to this suggestion; are you referring to virtual wards? | Lines 73-74  Replaced with “embed telehealth into clinical practice and increased adoption of telehealth platforms alongside facilitating integrated care, enhance service efficiency” |
| Line 74-75 you make a claim that “…VW programme exemplifies the solutions promoted by HSE’s ‘Telehealth Roadmap 2024-27’ strategy - please outline how VWs will address the suggested strategies included in the Roadmap. | Lines 79-81  Added a line “by enhancing accessibility and reducing care inequities, implementing digital solutions, improving patient safety and quality of care through digital technologies and ensuring data security and digital governance” |
| Line 78 and throughout the manuscript please use consistent terms or define roles such as “clinician” -how are you defining practitioners / clinicians? | Replaced all with ‘practitioners’ |
| Line 81-82 you state, “Studies aimed at understanding the attitudes of those at the centre of implementation have been emphasized as a crucial component of the implementation process…” Consider re-phrasing; most successful implementation projects acknowledge the importance of an interdisciplinary team. Clinicians would be an important stakeholder as the end user. | Lines 86-89  Replaced with “Research has emphasized that studying the attitudes of those directly involved in implementation is a crucial component of the process. Additionally, successful implementation projects recognize the importance of an interdisciplinary team, with clinicians being key stakeholders as end users.” |
| Line 94-95 Please clarify; do you mean that a study protocol was not generated as a separate manuscript? | Study protocol was not generated as a separate manuscript.  ‘Deleted the sentence to avoid redundancy’. |
| Line 142 Additional detail is needed for data analysis; how many researchers analyzed the data; what was your process to facilitate consistent analyses among researchers; how did you resolve differences; describe the background and experience among researchers contributing to the analysis - who had experience working with VW; what disciplines contributed (engineering, nurse, physician, IT...). Was the analysis inductive/deductive or both; did you have an initial code book to direct your analysis; did you use an implementation theory or framework to guide this analysis.... | Lines 150-156  Added a paragraph “The data were coded using NVivo by two researchers (TS and NR) under the supervision of WC, with input from JB, GM, and SL, who collaborated in refining themes. The study team (KM and NS) also included physicians involved in the implementation of AVWs in Ireland. To ensure consistency, TS and NR independently coded the data before discussing and refining themes with the broader team. Discrepancies were resolved through group discussion and consensus. The analysis followed an inductive approach.” |
| Line 163-164 Please provide clarity and revise; are you suggesting broadly that all staff (who is 'staff'?) reported satisfaction (satisfaction with what). And this was supported by one reference? | Lines 178-179  Amended “Research showed high job satisfaction and engagement among multidisciplinary staff (e.g. GPs, nurses, admin staff, etc.) involved in VW compared to traditional hospital settings” |
| Line 164-165 See my comment above; these statements need to be tempered / contextualized | Amended “Healthcare staff involved in VWs expressed enthusiasm for the continued development of VWs, highlighting the meaningful impact they felt in delivering care” |
| Line 181 “resource allocation” - what resources (do you mean people?)are you referring to? | Line 197  Resource allocation refers to human resources and funding for staffing and equipment. |
| Line 188-189 How did you know administration was supportive or not? Please review for clarity | Lines 205-109  Amended “Higher patient volumes reported in models where hospital administrators actively supported implementation, as evidenced by resource allocation, policy facilitation, and engagement in service development. Additionally, integrating patient perspectives and fostering broad patient awareness contributed to increased patient uptake.” |
| Line 204-207 you state: “AVWs offer system-wide benefits, such as reduced emissions, cost savings, and shorter hospital stays (18, 27, 34). However, concerns about climate impact from vehicle use persist, with recommendations for electric vehicles (18). Financial sustainability remains a challenge, requiring public funding and regional support”; these are broad statements that require additional description/ ‘evidence’ for readers to fully comprehend and appreciate the intended. | Lines 223-229  Replaced with  “VWs has the potential to reduce travel-related emissions of patients and staff by promoting greener models of healthcare with reduced hospital stays and alternative care models, allowing to deliver healthcare in the future of challenging weather events that might have impact on patient and staff mobility.”  “Financial sustainability remains a challenge due to inadequate funding for staff, equipment and home care services, requiring public funding and regional support.” |
| Line 215 regarding safety and the use of alarm devices resulting in increased confidence; please provide additional insight into ‘lone workers’ and the purpose of home visits within a VW model of care; please address whose confidence is impacted - the health care organization or the provider or both? What is the scope of this concern? | Lines 237-243  Amended “In a virtual care model, home visits are sometimes necessary for tasks such as physical assessments, equipment setup, blood work or urgent interventions that cannot be managed remotely. Staff safety concerns primarily revolved around home hazards and lone worker visits, where a single healthcare professional conducts these visits without immediate on-site support. Although lone worker alarm devices increased confidence among both providers and healthcare organizations, their limitations (e.g., not directly escalating to police) remain a concern regarding overall safety.” |
| Line 216-217 you state “Technology such as GPS tracking and recording devices may enhance safety”. Please consider the alternative that such tracking may elevate the experience of surveillance among providers or workers or … and this may have implications for enhanced safety but also for lack of privacy. | Lines 244-247  Added a line “However, effective implementation of a virtual care program should balance these benefits with concerns about privacy and the heightened sense of surveillance among service providers, ensuring that both physical safety and digital security of patients and staff are upheld.” |
| All results tables – please revise the title to include VW | Revised |
| Line 229-230 would you consider the clinical experience expressed here to be unique to only within VW models of care? | Lines 257-258  Added a sentence “ a challenge that can arise in any care model but was exacerbated by the virtual setting, causing frustration.” |
| Line 234 you claim ‘…the need for active patients and roles” ; provide additional detail to support reader comprehension of this claim – describe how patients need to be ‘active’ etc. | Lines 261-263  Replaced with “ increasing the need for patients to play an active role in their care rather than just lying in a hospital bed in the case of ED.” |
| Line 235 you “…more intimate environment…” ; do you mean their home context or lived environment? Please provide clarity | Lines 263-365  Refers to the home environment.  Replaced with “Staff appreciated the benefit of seeing patients in a home environment, helping them to understand the interplay between the home environment and symptoms better, providing additional context during care interactions” |
| Line 280-281 requires additional description/ information | Lines 313-317  Amended “Additionally, direct collaboration with hospital specialists, particularly cardiologists, played a critical role in promoting referrals to VW. This engagement included cardiologists providing clear referral criteria during multidisciplinary team meetings, conducting informational workshops for referrers, and offering ongoing case-based guidance to ensure appropriate patient selection.” |
| Line 311-312 consider caution in the broad statement / claim made here - the literature is limited, and this is based on one or few studies | Line 345  Replaced with “Studies included in this review” |
| Line 382 “methodological considerations” appears to be your limitations section – please revise. | Changed the title as ‘strengths and limitations’ |
